# Prioritizing competencies for soldier’s mental resilience: an application of integrative fuzzy-trapezoidal decision-making trial and evaluation laboratory in updating training program

**DOI:** 10.3389/fpsyg.2023.1239481

**Published:** 2024-02-05

**Authors:** Svajone Bekesiene, Rasa Smaliukienė, Ramutė Vaičaitienė, Dalia Bagdžiūnienė, Rosita Kanapeckaitė, Olena Kapustian, Oleksandr Nakonechnyi

**Affiliations:** ^1^General Jonas Zemaitis Military Academy of Lithuania, Vilnius, Lithuania; ^2^Taras Shevchenko National University of Kyiv, Kyiv, Ukraine

**Keywords:** cognitive skills, resilience training, experienced warriors, trapezoidal-fuzzy numbers, DEMATEL

## Abstract

**Background:**

The development of resilience is of the utmost importance in military training due to the demanding and high-stress nature of combat situations. Although there have been numerous studies on resilience competencies in the military, there is a research gap when it comes to identifying the most essential competencies that should be prioritized in training programs, particularly within compressed timeframes. With the current geopolitical landscape and ongoing military conflicts in Europe, it is necessary to expedite training of soldiers, including resilience training, without compromising the effectiveness of the program. This study aims to address this research gap by using a reductionist approach to resilience training and identifying the critical competencies that senior soldiers need to be trained to coach younger soldiers to maintain psychological strength during deployment. By filling this research gap, the study will contribute to the development of more efficient and targeted resilience training programs that optimize the ability of soldiers to adapt and excel in challenging military environments.

**Methods:**

To address the issue, this study assessed the competencies comprising the master resilience training (MRT) program, widely recognized as one of the most effective military resilience training programs. Two groups of military experts, totaling 16 individuals, were involved in the evaluation process, representing two military contexts. The first group consisted of Ukrainian military experts whose experiences primarily focused on defending their own country’s territory. The second group comprised Lithuanian military experts who had greater expertise in conducting military missions abroad. The assessment of resilience competencies was carried out using a deep analysis approach through the application of effective multi-criteria decision making (MCDM). Specifically, the decision-making trial and evaluation laboratory (DEMATEL) method was used, which is a significant multicriteria technique used to determine relationships among criteria and assign weight coefficients. In this study, the DEMATEL model was extended using trapezoidal fuzzy numbers (TrFN-DEMATEL) to accommodate decision-making under uncertainty conditions.

**Results:**

The research findings highlight the critical importance of three core resilience competencies: self-regulation, mental agility and strength of character. The importance of each competency varies depending on the specific military context. When defending one’s own country’s territory, strength of character emerges as the key factor in enhancing soldiers’ mental resilience. Conversely, during military operations abroad, self-regulation is the primary factor that promotes psychological resilience. Furthermore, the results show that these three primary competencies form a ‘cause group’ that influences other competencies through a cause-and-effect dependency.

**Conclusion:**

Based on the findings, the theoretical conclusion is drawn that the importance of resilience competencies is contextually differentiated. Furthermore, each resilience competency is associated with a set of causes or effects. These are valuable insights for improving resilience competency training programs.

## 1 Introduction

Building resilience is a crucial aspect of military training that enhancing soldiers’ capacity to adapt to combat stress. While many military resilience training programs aim to develop a range of competencies relevant to building resilience, the current geopolitical landscape and ongoing military conflicts in Europe underscore the need for expedited solder training, including resilience training. In the face of compressed training timeframes for new deployments, it becomes essentials to adopt a reductionist approach to resilience training program, focusing on the most critical competencies for success in combat situations and military life. By adopting this approach, we can ensure that soldiers receive the necessary training to develop the resilience required to excel in their service.

Numerous studies have examined building resilience competencies in the military environment, many of these studies rooted in positive psychology theory. This theory posits that resilience is an individual’s ability to adapt positively to stressful situations (Masten et al., 2009). Positive adaptation is based on two key assumptions: first, that the individual has experienced high levels of adversity, and second, that the individual responds positively in their own interest when exposed to such situations (Deppa and Saltzberg, 2016a). Both positive adaptation and responses to unfavorable circumstances are central to the definition of resilience (Luthar et al., 2000). In the military context, resilience prevents from the adverse effects of combat deployment, which can cause various mental disorders, including post-traumatic personality transformation (Thomsen et al., 2011; McInerney et al., 2022) as well as increases soldiers long-term commitment to the military organization (Bekesiene et al., 2023a). Consequently, selecting the competencies that would have the greatest impact on resilience and developing these competencies during training could significantly shorten the pre-deployment training without jeopardizing the ability to withstand the adverse psychological effects.

To address this issue, we conducted a revision of the competences included in the master resilience training (MRT) program. Developed from the principles of positive psychology and thoroughly tested in military settings (Reivich et al., 2011), MRT is regarded as one of the most effective military resilience training programs available today (McInerney et al., 2022). This is a train-the-trainer program where senior soldiers are trained to help juniors ones by focusing and developing six groups of competencies: self-awareness, self-regulation, optimism, mental agility, strengths of character, and connection (MRT Skills Overview, 2014). Each competence developed using several prophylactic interventions (Carr et al., 2013). For example, to grow self-awareness, the senior solder encourages junior ones to reflect on their experiences, both positive and negative, provides feedback and encourages them to seek feedback from others, teaches soldiers mindfulness techniques and develops their emotional intelligence skills, such as recognizing and managing their own emotions (MRT Skills Overview, 2014).

As one of the most extensively utilized resilience development program in the military, specifically in the army, MRT program underwent testing in a various setting. Harms et al. (2013) conducted a quasi-experimental longitudinal study involving a large-scale group of participants to assess the impact of MRT instructors on the self-perceived resilience of US Army soldiers within combat units. The findings revealed that the resilience training conducted by the MRT trainers within the units indirectly improved the mental health of the soldiers by promoting greater optimism and adaptability. Moreover, the study identified a negative association between training and psychoactive substance abuse. Another study, conducted by Lester et al. (2011a,b) employed a similar methodology and spanned 15 months. This study demonstrated significantly higher levels of soldier-reported resilience and psychological health in groups with MRT trainers compared to control groups without MRT trainers. The observed difference between the affected and control groups was equal to or greater than observed in other resilience—building programs proven effective in a civilian context. Importantly, the effects of MRT were analyzed under various conditions, including training and combat deployment contexts. Additionally, this study found that MRT training exhibited greater effectiveness among younger soldiers compared to older ones. These large-scale participant studies conclude that an MRT program, which incorporates mentor deployment and their presence within units, can effectively reduce the incidence of mental health issues among soldiers (Lester et al., 2011a,b; Harms et al., 2013). However, other research has produced less favorable results. A study conducted on soldiers deployed in Afghanistan initially reported a positive correlation between the implementation of this program and self-reported positive resilience thinking and morale; nevertheless, over time, both the resilient mindset and morale exhibited a decline (Carr et al., 2013).

The effectiveness of this program has been assessed in various countries and contexts that extend beyond the military. For instance, when MRT was applied to students, it was observed that the training had a positive impact on anxiety reduction, as measured by the Zung anxiety scale and SCL-90 (Ambrosio and Adiletta, 2021). Additionally, participants demonstrated improved social interactions after training. The discussion on the value of MRT elements, such as optimism, mental agility, and social connections, emphasized their significance in developing resilience among firefighters (Deppa and Saltzberg, 2016b).

Considering the program’s several decades of use, multiple proposals have been made to modify it. For instance, during a military medical personnel training research, a suggestion was made to eliminate certain components of the program (Start et al., 2017). The content of the MRT program underwent testing on Army National Guard soldiers, who typically hold full-time jobs in non-military environments. The research results indicate that if the content is adopted in accordance with the principles of adult learning theory, positive outcomes are observed, particularly in terms of perceived resilience, goal setting application, and emotional control (Howard et al., 2022).

Despite the lack of theoretical underpinnings in the MRT construct, its effectiveness has been repeatedly validated by empirical studies (McInerney et al., 2022). However, it is worth considering the possibility of reducing the competencies included in this model, as they may exhibit interdependencies. This trend aligns with other resilience models proposed by academics, who have advocated for a reduction in competencies. For example, during the Covid pandemic, it was found that effective recovery from stress response and positive assessment were the two most influential factors for resilience in different countries, highlighting their significance amidst the specific stressors posed by the pandemic (Veer et al., 2021). Furthermore, most studies that evaluate the effectiveness of MRT have focused on immediate post-training evaluation. Nonetheless, as pointed by van der Meulen et al. (2018) here exists a substantial disparity between short-term and long-term effects of resistance training. It is plausible that only certain competencies remain relevant in the long run.

Given the limited training time for senior soldiers to act as resilience trainers and the challenge of providing personalized attention to younger soldiers during deployment, we recognized the need to streamline the list of competencies. By focusing on the most essential competencies, we can maximize the effectiveness of the training program. For this purpose, we gathered expert survey data from experienced military professionals from Ukraine and Lithuania to identify the critical competencies needed for soldiers to maintain resilience during combat situations of indefinite duration, as well as for timed missions. To analyze the data, we employed the techniques of fuzzy logic, which is designed to obtain accurate results even when the information is imprecise or ambiguous, relying on heuristic methods such as experts’ surveys.

The aim of this study is to identify the critical resilience competencies that senior soldiers need to be trained to coach younger soldiers to maintain psychological strength during deployment. To achieve this, we utilized the decision making and trial evaluation laboratory (DEMATEL) method. Using this method, we were able to assess the interrelationships between different competencies and determine which ones have the greatest impact on overall resilience. The DEMATEL method distinguishes complex factors into cause and result groups and generates a visual cause-and-effect relationship diagram, providing an effective way to find countermeasures and make decisions about complex problems (Bekesiene et al., 2022b). The study employs the fuzzy DEMATEL method, utilizing trapezoidal fuzzy numbers to develop a causal diagram of resilience competencies and prioritize them based on their level of importance.

## 2 Literature review focused on soldiers’ resilience competencies training

The literature review enabled us to identify that various training programs have been developed with the aim to enhance soldiers’ resilience. These resilience trainings primary concentrate on improving mental health outcomes and aim to promote psychological resilience among service members through implementation of diverse strategies. These trainings can be characterized as preventive interventions. For instance, one such program, the Army Center for Enhanced Performance (ACEP), focuses on building up the mind-body connection through six components grounded in applied sport, health, and social psychology, which have the potential to enhance soldiers’ performance (Dibble, 2015). Another prominent military resilience training program, known as Battlemind training (Castro et al., 2006), is designed to provide comprehensive mental training based on a range of psychological theories. Additionally, the well-known mindfulness-based mind fitness training (Stanley, 2014) incorporates targeting the structure and functioning of the soldier’s brain, serving as a protective measure for their mental health.

Another extensively researched resilience competence training approach is provided by The U.S. Army Master Resilience Trainer (MRT) program, which was developed by the University of Pennsylvania as a part of Penn resilience program (PRP) (Reivich et al., 2011). The MRT program follows the “train the trainer” methodology and all the trainings lasts 10 days. Teaching process goes on as face-to-face resilience exercise training. MRT comprises of three modules: (1) preparation, (2) sustainment, and (3) enhancement; and is considered as one of the foundational components of the all-inclusive soldier competence program.

During MRT training, sergeants are instructed how to enhance soldiers’ key resilience competencies such as: self-awareness (C1); self-regulation (C2); optimism (C3); mental agility (C4); strength of character (C5); and connection (C6):

- Self-awareness (C1) competence aids soldiers in better understand their strengths, weaknesses, and helps in coping with stress and adversity, as well as helps making better decisions in high-pressure situations (Crane et al., 2019; Schrader, 2019; Safran et al., 2022). By developing self-awareness, soldiers can become more effective and resilient improving their ability to handle the demands and challenges of military service. Skills that can enhance soldier’s self-awareness include: (1) reflective practice; (2) feedback, (3) mindfulness, (4) emotional intelligence:
- Self-regulation (C2) competence involves effectively managing one’s thoughts, emotions, and behaviors effectively in response to different stressful situations (Gün, 2011; Gibbons et al., 2012; Murray and Ehlers, 2021). It is a critical skill for soldier to possess this ability as they face various challenging and stressful situations in their line of duty. Soldiers can enhance self-regulation by rising key skills: (1) identifying triggers; (2) developing coping strategies; (3) practicing self-reflection; (4) setting realistic goals; and (5) seeking support when needed.
- Optimism (C3) competence is an important quality for soldiers to possess, as it helps maintaining a positive attitude and outlook even in challenging situations (Seligman and Csikszentmihalyi et al., 2014; Crabtree-Nelson and DeYoung, 2017). Optimistic soldiers are more likely to persevere and find solutions to problems, believing that things will ultimately work out for the best. Key skills to improve optimism: (1) focus on the mission; (2) develop a positive mindset; (3) seek support; and (4) stay resilient.
- Mental agility (C4) competence is an essential for a soldier’s performance in various situations (Ashworth et al., 2020; Smith et al., 2022; Bekesiene et al., 2023b). It refers to the ability to process information, think critically, and make decisions in high-pressure environments quickly and accurately. Soldiers can improve mental agility through (1) practices mindfulness; (2) physical exercise; (3) mental exercises; (4) improving communication skills; and (5) seeking professional help.
- Strengths of character (C5) is a positive personality trait and quality (Heřman et al., 2022). Soldiers of character may have an advantage in performing their duties effectively. Key skills to improve strengths of character includes: (1) courage; (2) perseverance; (3) self-discipline; (4) honesty; (5) teamwork; and (6) compassion.
- Connections (C6) are important for soldiers, as they can provide support, encouragement, and a sense of friendship during the challenges of military life (Bowles et al., 2015; Wang et al., 2015; Williams-Klotz and Gansemer-Topf, 2018). Building strong connections within and outside of the military are essential for soldiers to thrive. Soldiers can build a strong support network that can help them navigate the challenges of military life and achieve their goals by fostering connections with their unit, family and friends, community.

Accordingly, MRT program aims to enhance cognitive and social skills by incorporating empirically confirmed insights from positive psychology (Peterson and Seligman, 2004), and promoting the development of strong relationships (Gable et al., 2004). The enhancement of these six resilience competencies is vital for deployment and life cycles throughout soldiers’ careers. The inclusion of these competencies and skills in the training program is supported by existing research literature (see Table 1).

**Table 1:** Literature supporting the validity of soldier resilience training.

Competence	Description of skills to be developed	Research authors
Self-awareness (C1)	(1) Reflective practice. Encourage soldiers to reflect on their experiences, both positive and negative. This can help them to identify patterns of behavior and thought, and to better understand their reactions to different situations	Reivich et al. (2011) and Crane et al. (2019)
	(2) Feedback. Provide soldiers with feedback on their performance, and encourage them to seek feedback from others. This can help them to identify areas where they need to improve, as well as areas where they excel	Cornum et al. (2011) and Binsch et al. (2017)
	(3) Mindfulness. Teach soldiers mindfulness techniques, such as meditation or deep breathing exercises, to help them stay focused and calm in stressful situations. Mindfulness can also help them to become more self-aware by bringing their attention to their thoughts, emotions, and physical sensations	Reivich et al. (2011) and Safran et al. (2022)
	(4) Emotional intelligence. Help soldiers develop emotional intelligence skills, such as recognizing and managing their own emotions, as well as understanding and empathizing with others. This can help them to better navigate interpersonal relationships, communicate effectively, and make better decisions	Aguilar and George (2019) and Garcia Zea et al. (2019)
Self-regulation (C2)	(1) Identifying triggers. Soldiers need to understand what triggers their emotional responses and behaviors. They can keep a journal or talk to a mental health professional to help them identify their triggers	Murray and Ehlers (2021)
	(2) Developing coping strategies. Once soldiers have identified their triggers, they can develop coping strategies to manage their emotions and behavior. Coping strategies can include deep breathing, visualization, physical exercise, and mindfulness techniques	Reivich et al. (2011) and Delahaij and van Dam (2016)
	(3) Practicing self-reflection. Soldiers can practice self-reflection to identify their strengths and weaknesses in self-regulation. They can take time to reflect on their actions, emotions, and behaviors and make changes where necessary	Gün (2011) and Reivich et al. (2011)
	(4) Setting realistic goals. Setting realistic goals can help soldiers manage their emotions and behavior effectively. They can break down larger goals into smaller, achievable ones and celebrate their progress along the way	Reivich et al. (2011) and Gibbons et al. (2012)
	(5) Seeking support. Soldiers can seek support from their peers, leaders, or mental health professionals when they need it. Talking to someone about their emotions and behaviors can help soldiers manage them effectively	Reivich et al. (2011) and Hom et al. (2017)
Optimism (C3)	(1) Focus on the mission. Soldiers who maintain a strong focus on their mission and the goals they are working towards are more likely to stay motivated and optimistic, even in the face of obstacles	Demetriou and Schmitz-Sciborski (2011)
	(2) Develop a positive mindset. Encouraging positive self-talk, practicing gratitude, and surrounding oneself with positive influences can all help to cultivate a more optimistic mindset	Reivich et al. (2011) and Seligman Csikszentmihalyi et al. (2014)
	(3) Seek support. Soldiers who have a strong support network, both within their unit and outside of it, are better equipped to handle the challenges of military life and maintain a positive outlook	Reivich et al. (2011)
	(4) Stay resilient. Resilience is the ability to bounce back from setbacks and difficult situations. Soldiers who develop strong resilience skills are better able to maintain a positive outlook, even in the face of adversity	Reivich et al. (2011) and Crabtree-Nelson and DeYoung (2017)
Mental agility (C4)	(1) Practice mindfulness. Mindfulness can help soldiers remain focused and present in the moment. It can also help them manage stress and anxiety, which can affect their mental agility. Mindfulness exercises like deep breathing, meditation, and visualization can be helpful	Zimmermann (2015)
	(2) Engage in physical exercise. Physical exercise can help increase blood flow to the brain, which can enhance cognitive function. Soldiers can engage in activities like running, weightlifting, and other forms of physical exercise to improve their mental agility	Ashworth et al. (2020)
	(3) Participate in mental exercises. Mental exercises like puzzles, brain teasers, and memory games can help improve cognitive function and enhance mental agility. Soldiers can also engage in simulation exercises that mimic real-life scenarios to improve their decision-making abilities	Pargament and Sweeney (2011) and Summers (2012)
	(4) Improve communication skills. Communication is an essential aspect of military operations, and soldiers who can communicate effectively can make better decisions in high-pressure situations. Soldiers can improve their communication skills by practicing active listening, speaking clearly and concisely, and giving and receiving feedback	Brathwaite (2018)
	(5) Seek professional help. Soldiers who experience mental health challenges like anxiety, depression, or post-traumatic stress disorder (PTSD) should seek professional help. Mental health professionals can provide soldiers with the support they need to overcome these challenges and improve their mental agility	Schneider et al. (2023)
Strength of character (C5)	(1) Courage. Courage allows soldiers to face danger, fear, and uncertainty with bravery and determination	Goud (2005)
	(2) Perseverance. Perseverance allows soldiers to endure and persist through difficult and challenging situations. It helps them maintain focus and determination even in the face of adversity	Sousa and Hagopian (2011)
	(3) Self-discipline. Self-discipline is the ability to control one’s behavior, emotions, and impulses. Soldiers who possess self-discipline can follow orders, maintain composure, and avoid distractions that may compromise their performance	Wilson (2014)
	(4) Honesty. Honesty is a vital strength of character for soldiers. It allows them to maintain integrity and uphold their ethical standards even in challenging situations	Gayton and Kehoe (2015) and Dobbs et al. (2019)
	(5) Teamwork. Soldiers need to work together effectively to achieve their goals. Teamwork allows soldiers to collaborate, communicate effectively, and support each other to achieve mission success	McGurk et al. (2006)
	(6) Compassion. Compassion is the ability to understand and empathize with others. Soldiers who possess compassion can provide support and care to their fellow soldiers, even in stressful and challenging situations	Gayton and Kehoe (2015)
Connection (C6)	(1) Connection to UNIT. Soldiers who feel a strong sense of connection and belonging to their unit are more likely to perform well and have a positive experience in the military. This connection can be fostered through team-building activities, training exercises, and shared experiences	McGurk et al. (2006)
	(2) Connection to family and friends. Maintaining connections with family and friends outside of the military can provide soldiers with a sense of support and stability. Regular communication and visits with loved ones can help soldiers stay connected to their civilian lives and maintain a sense of balance	Riggs and Riggs (2011), Hall (2012) and Bowles et al. (2015)
	(3) Connection to community. Soldiers who feel connected to their community, whether it be through volunteer work or participation in local events, may experience a greater sense of purpose and belonging outside of the military	Wang et al. (2015) and Williams-Klotz and Gansemer-Topf (2018)
	(4) Connection to mental health resources. Soldiers who have access to mental health resources and support are better equipped to deal with the unique stresses and challenges of military life	Masten and Obradovic (2008)

This literature review has revealed that the military resilience training programs primary focuses on enhancing mental health outcomes and foster psychological resilience that is vital for deployment and life cycles throughout soldiers’ careers. The aforementioned programs are specially designed to enhance solders’ psychological performance and mitigate mental health issues in a preventive manner. However, the effectiveness of completed resilience program is often limited in terms of evaluating actual long-lasting changes in the targeted behavior; instead, the emphasis is often placed on the quantity of training attendance or on sort-term effect rather than evaluation the desired behavioral change (Lester et al., 2011a,b). Furthermore, various resilience competencies training programs for solders tend to focus on improving different aspects of resilience; their effectiveness evaluation typically relies on self-reported questionnaires (Castro et al., 2012). While these evaluations contribute to a better understanding of the significance of resilience competencies, they also pose limitations in terms of comparability of competencies developed. To address this limitation, it is necessary to reevaluate resilience competencies and explore their bidirectional relationship to identify the most valuable ones that yields the greatest benefits.

## 3 Methodology

### 3.1 The DEMATEL application to optimize a list of competences

DEMATEL is designed to analyze and visualize the relationships of complex cause-and-effect models using matrices and graphs (Si et al., 2018). This is especially useful in decision-making when deciding which competencies (factors) are essential for growth during training to achieve the desired mental resilience. Graphs and network maps make it easier to understand the relationships between factors and make decisions about which factors to further modify or strengthen (Ullah et al., 2021). Factors are evaluated according to criteria, so in DEMATEL criteria are ranked according to the type and importance of the interrelationships (Cebi, 2013). Criteria that have a greater influence on others are classified in the “cause” group, while those that are influenced by others are classified in the “effect” groups; using these two groups, the interdependence of the criteria is identified and translated into a cause-effect structural model (Ullah et al., 2021).

**Figure 1:**
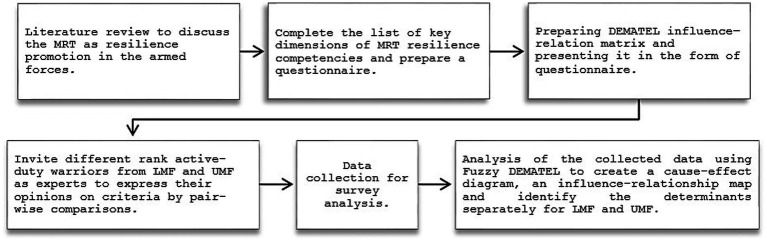
The steps of conducted investigations presented by scheme.

In our study, the application of the DEMATEL method not only facilitates the categorization of complex factors into cause-and-effect groups, but also addresses the bidirectional relationship among resilience competencies. The DEMATEL method offers a solution to this issue by examining the relationships between selected dimensions and factors. The determination of factors influencing resilience has been extensively examined in psychological theory, and various competence models have been proposed. However, the challenge arises from the interconnected nature of resilience competencies. For instance, research by Morosanova et al. (2021) highlights the positive influence of optimism on self-regulation, whereas Wang et al. (2022) suggest that optimism and social support reveal the effects of goal-oriented self-regulatory behavior. Similarly, the relationship between mental agility and strengths of character exhibits reciprocity. Empirical findings indicate that mental agility is influenced by the complexity of cognitive demands (Büchel et al., 2022), which, in turn, are shaped by strengths of character. Conversely, Hosein and Yousefi (2012) investigate the inverse relationship, exploring how self-control impacts agility. Therefore, in the binary relationship between resilience competencies, either factor can influence the other. Given that the MRT instrument comprises six competencies, which are further composed of factors, we were able to bypass the initial step of factor grouping and proceed directly to the second step. The second step involved developing a questionnaire for the paired evaluation of criteria, and Ukrainian and Lithuanian army experts meeting the study’s criteria were invited to express their opinions on resilience factors. Moving on to the third step, we employed the fuzzy technique with DEMATEL (Pribićević et al., 2020) to examine and assess the ambiguous and indefinite nature of military psychological resilience. This comprehensive methodology allowed for the study of the multidimensional and interactive nature of military resilience, with fuzzy theory used to convert expert assessments of semantic resilience factors into evaluator’s degree value through the membership function utilizing trapezoidal fuzzy numbers. Finally, the modeling results were presented in two diagrams: a cause-and-effect diagram and an influence-relations map. Additionally, for more clarity, the steps of this study are explained in a diagram (see Figure 1).

### 3.2 The study procedure

Sample. At the core of the research plan was the strategic engagement of experts from diverse military contexts, as the aim of this study was to prioritize competencies for soldiers’ mental resilience in diverse military contexts. To achieve this, two groups of experts were engaged during the data collection phase. The first group consisted of eight experts from the Ukrainian Armed Forces (UMF), whose expertise focused on enhancing the resilience of soldiers defending their homeland. These experts were carefully selected on the basis of their professional competence, in particular their experience in mental resilience training for front-line soldiers, as well as their length of service in the military. The second group consisted of eight military experts from the Lithuanian Military Forces (LMF) with extensive experience in resilience of soldiers on international military missions abroad. The selection criteria for these experts included their expertise in resilience building, military service and completion of international missions. Detailed information on the experience of the experts and the missions carried out is not given here due to information constraints.

For the purpose of this research, a cohort of 16 experts was interviewed. The experts selected for this study were from regions with distinct geopolitical situations, specifically Ukraine and Lithuania. Recognizing that these unique circumstances might have influenced the opinions of the study experts, they were divided into two groups. The study employed a pairwise comparison questionnaire to conduct an in-depth analysis and to comprehend the substantial divergence in opinions between Ukrainian and Lithuanian experts. The research instrument consisted of six MRT competencies (Reivich et al., 2011; Griffith and West, 2013; MRT Skills Overview, 2014) with their short description of common cognitive behaviors that soldiers may exhibit:

- Self-awareness (C1). A soldier recognizes unproductive thoughts and emotions, especially in critical situations, and understands that different behaviors are productive in different situations.
- Self-regulation (C2). A soldier maintains emotional control and remains calm in stressful situations. This helps them make rational decisions and avoid impulsive or reckless choices that could put themselves or others in danger.
- Optimism (C3). A soldier maintains rational optimism even in difficult situations, trusting in himself and the team.
- Mental agility (C4). A soldier is able to adapt quickly to changing situations and make decisions under pressure. This requires flexibility and the ability to think on one’s feet.
- Character strengths (C5). A soldier performs effectively because he knows his character strengths and the skills and abilities he possesses to overcome challenges and achieve goals.
- Connection (C6). Soldiers often work in teams, and teamwork is essential to accomplishing tasks effectively. Soldiers are trained to communicate effectively, work cooperatively, and support their teammates.

The instrument was translated from English into Lithuanian and Ukrainian. The translation was evaluated by teams of bilingual psychologists. The study was conducted in 2023 by researchers at the Military Academy of Lithuania.

Furthermore, the trapezoidal fuzzy number (TrFN-DEMATEL) method was conducted by eight steps. To perform this analysis, the opinions on six psychological resilience competencies were collected by filling out a pairwise comparison questionnaire from eight Ukrainian and 10 Lithuanian qualified soldiers as military resilience experts on the field. The opinions of the study experts were expressed by linguistic terms that were presented in the set of prepared linguistic terms (see Table 1). Therefore, the pairwise judgement of six resilience competencies was evaluated by scores of nine linguistic terms, such as: S1 = “EXL,” S2 = “VLI,” S3 = “LI,” S4 = “MLI,” S5 = “MI,” S6 = “MHI,” S7 = “HI,” S8 = “VHI,” S9 = “EXH” which were associated with positive trapezoidal-fuzzy numbers (see Table 1). These two collected data sets (1) Ukrainian experts and (2) Lithuanian experts allow us to conduct eight steps of fuzzy trapezoidal DEMATEL method and identify complex causal relationships among the six soldiers’ resilience competencies: C1 (self-awareness), C2 (self-regulation), C3 (optimism), C4 (mental agility), C5 (character strengths), and C6 (connection), for Ukrainian and Lithuanian soldiers in a separate mode.

## 4 Empirical study results

### 4.1 Establishing a direct-relation matrix

As a first step, the direct relationship matrix D was prepared for our investigations and the comprehensive evaluation procedure was conducted using the TrFN-DEMATEL investigation steps (see Supplementary material). The two direct relationship matrices D were designed for six main resilience competencies in case to analyze in separate mode the judgements of Ukraine and Lithuanian soldiers, who were chosen as experts for this study. The aggregated experts’ opinions on the importance of six soldiers’ resilience competencies noted for this study are presented in linguistic terms in Table 2.

**Table 2:** The averaged expressed experts’ decision to show the importance of soldiers’ resilience competencies.

DM1							DM2						
	C1	C2	C3	C4	C5	C6		C1	C2	C3	C4	C5	C6
C1	0	M	H	H	M	M	C1	0	VL	MH	VL	EL	EL
C2	MH	0	H	M	VH	M	C2	VH	0	VH	ML	MH	VH
C3	M	ML	0	L	L	M	C3	VL	VL	0	VL	VL	ML
C4	MH	MH	H	0	MH	H	C4	VH	ML	VH	0	M	VH
C5	H	H	MH	M	0	H	C5	EL	L	M	ML	0	MH
C6	H	M	L	MH	ML	0	C6	EL	VL	M	VL	L	0

DM1 = aggregated Ukraine experts’ judgement; DM2 = aggregated Lithuania experts’ judgement. C1 = self-awareness, C2 = self-regulation, C3 = optimism, C4 = mental agility, C5 = strengths of character, and C6 = connection. Data expressed in linguistic terms: VL, very low; L, low; M, medium influence; H, high influence; VH, very high influence.

To continue with the study procedure, the linguistic terms in direct-relation matrices were changed into trapezoidal fuzzy numbers following the fuzzy semantic measure and its equivalent fuzzy value, the attribution function. Consequently, the guidance recognized by the linguistic variable was changed into a positive trapezoidal fuzzy number taking into account the values represented in Supplementary Table S1. In this way, the initial fuzzy direct-relation matrix D was gained. The initial direct relationship matrix constructed that separately represents the judgments of Ukrainian and Lithuanian experts on six soldiers’ resilience competencies are shown in Table 3.

**Table 3:** The initial direct relation matrix representing both Ukrainian and Lithuanian experts’ judgement.

DM1	C1	C2	C3	C4	C5	C6
C1	(0, 0, 0, 0)	(4, 5, 6, 7)	(6, 7, 8, 9)	(6, 7, 8, 9)	(4, 5, 6, 7)	(4, 5, 6, 7)
C2	(5, 6, 7, 8)	(0, 0, 0, 0)	(6, 7, 8, 9)	(4, 5, 6, 7)	(7, 8, 9, 10)	(4, 5, 6, 7)
C3	(4, 5, 6, 7)	(1, 2, 3, 4)	(0, 0, 0, 0)	(2, 3, 4, 5)	(2, 3, 4, 5)	(4, 5, 6, 7)
C4	(5, 6, 7, 8)	(5, 6, 7, 8)	(6, 7, 8, 9)	(0, 0, 0, 0)	(5, 6, 7, 8)	(6, 7, 8, 9)
C5	(6, 7, 8, 9)	(6, 7, 8, 9)	(5, 6, 7, 8)	(4, 5, 6, 7)	(0, 0, 0, 0)	(6, 7, 8, 9)
C6	(6, 7, 8, 9)	(4, 5, 6, 7)	(2, 3, 4, 5)	(5, 6, 7, 8)	(3, 4, 5, 6)	(0, 0, 0, 0)
C1	(0, 0, 0, 0)	(1, 2, 3, 4)	(5, 6, 7, 8)	(1, 2, 3, 4)	(0, 1, 2, 3)	(0, 1, 2, 3)
C2	(7, 8, 9, 10)	(0, 0, 0, 0)	(7, 8, 9, 10)	(3, 4, 5, 6)	(5, 6, 7, 8)	(7, 8, 9, 10)
C3	(2, 3, 4, 5)	(1, 2, 3, 4)	(0, 0, 0, 0)	(1, 2, 3, 4)	(1, 2, 3, 4)	(3, 4, 5, 6)
C4	(7, 8, 9, 10)	(3, 4, 5, 6)	(7, 8, 9, 10)	(0, 0, 0, 0)	(4, 5, 6, 7)	(7, 8, 9, 10)
C5	(0, 1, 2, 3)	(2, 3, 4, 5)	(4, 5, 6, 7)	(3, 4, 5, 6)	(0, 0, 0, 0)	(5, 6, 7, 8)
C6	(0, 1, 2, 3)	(1, 2, 3, 4)	(4, 5, 6, 7)	(1, 2, 3, 4)	(2, 3, 4, 5)	(0, 0, 0, 0)

DM1 = aggregated Ukraine experts’ judgement; DM2 = aggregated Lithuania experts’ judgement.

### 4.2 Calculating normalized direct-relation matrix

To continue with the study procedure, the normalized fuzzy directed-relation matrices were built. The transformation was carried out following the equations (8a) to (8d) and equation (9) for the identification of the maximum value and for all values in the calculation of the fuzzy direct-relation matrix (see Supplementary material). The normalized fuzzy direct-relation matrices are presented for Ukrainian and Lithuanian experts in Table 4.

**Table 4:** The normalized fuzzy directed-relation matrix.

DM1	C1	C2	C3
C1	(0.000, 0.000, 0.000, 0.000)	(0.029, 0.036, 0.043, 0.051)	(0.043, 0.051, 0.058, 0.065)
C2	(0.036, 0.043, 0.051, 0.058)	(0.000, 0.000, 0.000, 0.000)	(0.043, 0.051, 0.058, 0.065)
C3	(0.029, 0.036, 0.043, 0.051)	(0.007, 0.014, 0.022, 0.029)	(0.000, 0.000, 0.000, 0.000)
C4	(0.036, 0.043, 0.051, 0.058)	(0.036, 0.043, 0.051, 0.058)	(0.043, 0.051, 0.058, 0.065)
C5	(0.043, 0.051, 0.058, 0.065)	(0.043, 0.051, 0.058, 0.065)	(0.036, 0.043, 0.051, 0.058)
C6	(0.043, 0.051, 0.058, 0.065)	(0.029, 0.036, 0.043, 0.051)	(0.014, 0.022, 0.029, 0.036)
	**C4**	**C5**	**C6**
C1	(0.043, 0.051, 0.058, 0.065)	(0.029, 0.036, 0.043, 0.051)	(0.029, 0.036, 0.043, 0.051)
C2	(0.029, 0.036, 0.043, 0.051)	(0.051, 0.058, 0.065, 0.072)	(0.029, 0.036, 0.043, 0.051)
C3	(0.014, 0.022, 0.029, 0.036)	(0.014, 0.022, 0.029, 0.036)	(0.029, 0.036, 0.043, 0.051)
C4	(0.000, 0.000, 0.000, 0.000)	(0.036, 0.043, 0.051, 0.058)	(0.043, 0.051, 0.058, 0.065)
C5	(0.029, 0.036, 0.043, 0.051)	(0.000, 0.000, 0.000, 0.000)	(0.043, 0.051, 0.058, 0.065)
C6	(0.036, 0.043, 0.051, 0.058)	(0.022, 0.029, 0.036, 0.043)	(0.000, 0.000, 0.000, 0.000)
C1	(0.000, 0.000, 0.000, 0.000)	(0.007, 0.014, 0.021, 0.027)	(0.034, 0.041, 0.048, 0.055)
C2	(0.048, 0.055, 0.062, 0.068)	(0.000, 0.000, 0.000, 0.000)	(0.048, 0.055, 0.062, 0.068)
C3	(0.014, 0.021, 0.027, 0.034)	(0.007, 0.014, 0.021, 0.027)	(0.000, 0.000, 0.000, 0.000)
C4	(0.048, 0.055, 0.062, 0.068)	(0.021, 0.027, 0.034, 0.041)	(0.048, 0.055, 0.062, 0.068)
C5	(0.000, 0.007, 0.014, 0.021)	(0.014, 0.021, 0.027, 0.034)	(0.027, 0.034, 0.041, 0.048)
C6	(0.000, 0.007, 0.014, 0.021)	(0.007, 0.014, 0.021, 0.027)	(0.027, 0.034, 0.041, 0.048)
	**C4**	**C5**	**C6**
C1	(0.007, 0.014, 0.021, 0.027)	(0.000, 0.007, 0.014, 0.021)	(0.000, 0.007, 0.014, 0.021)
C2	(0.021, 0.027, 0.034, 0.041)	(0.034, 0.041, 0.048, 0.055)	(0.048, 0.055, 0.062, 0.068)
C3	(0.007, 0.014, 0.021, 0.027)	(0.007, 0.014, 0.021, 0.027)	(0.021, 0.027, 0.034, 0.041)
C4	(0.000, 0.000, 0.000, 0.000)	(0.027, 0.034, 0.041, 0.048)	(0.048, 0.055, 0.062, 0.068)
C5	(0.021, 0.027, 0.034, 0.041)	(0.000, 0.000, 0.000, 0.000)	(0.034, 0.041, 0.048, 0.055)
C6	(0.007, 0.014, 0.021, 0.027)	(0.014, 0.021, 0.027, 0.034)	(0.000, 0.000, 0.000, 0.000)

DM1 = aggregated Ukraine experts’ judgement; DM2 = aggregated Lithuania experts’ judgement.

### 4.3 Calculating total-relation matrix

After obtaining the normalized fuzzy direct-relation matrix and continuing study analysis, the total fuzzy directed-relation matrices $\tilde{G}$ were created following the equations (10), (11) and from (12a) to (12d) (see Supplementary material). Consequently, all fuzzy directed-relation matrices $\tilde{G}$ were defuzzified and all fuzzy values were transformed into crisp numbers as shown in Table 5.

**Table 5:** The defuzzied total-relation matrix into a crisp total-relation matrix.

DM1							DM2						
	C1	C2	C3	C4	C5	C6		C1	C2	C3	C4	C5	C6
C1	0.013	**0.049**	**0.065**	**0.063**	**0.050**	**0.051**	C1	0.004	0.019	**0.048**	0.019	0.013	0.015
C2	**0.059**	0.012	**0.066**	**0.050**	**0.070**	**0.052**	C2	**0.063**	0.006	**0.068**	**0.036**	**0.050**	**0.066**
C3	**0.047**	0.025	0.008	0.033	0.032	0.047	C3	0.027	0.019	0.006	0.019	0.020	**0.034**
C4	**0.060**	**0.057**	**0.066**	0.013	**0.057**	**0.065**	C4	**0.063**	**0.035**	**0.068**	0.006	**0.043**	**0.065**
C5	**0.066**	**0.064**	**0.059**	**0.051**	0.013	**0.065**	C5	0.015	0.027	**0.044**	**0.034**	0.005	**0.050**
C6	**0.064**	**0.048**	0.037	**0.056**	0.042	0.011	C6	0.014	0.019	**0.042**	0.020	0.027	0.005

DM1 = Ukraine experts’ judgement; DM2 = Lithuania experts’ judgement. This study applied the mean average as the threshold value: DM1 threshold is 0.047; DM2 threshold is 0.031.

The values in total relation matrices can be used to identify the common connections between six resilience competencies, but to clarify the relationships and eliminate unclear view on the influence-relations map, additionally, the threshold number of defuzzied total-relation matrix must be calculated. These calculations were performed and the threshold value for Ukrainian experts (0.047) and for Lithuanian experts (0.031) was identified individually.

### 4.4 Computing the centrality (D + R) and causality degree (D − R)

To continue the sequence of these study steps, the uncertain variance and correlation of resilience competencies were individually determined as the sum of each row (Ri) and each column (Ci) of the total relationship matrix using the mathematical equations from (13a) to (13d) (see Supplementary material). The calculation results are presented in Table 6 (see column Ri and Ci).

**Table 6:** The degree of centrality (R + C) and causality (R − C).

DM1 Competence	Ri	Ci	Ri + Ci	Ri − Ci	Identity	Rank
C1	0.291	0.309	0.601	−0.018	Effect	4
C2	0.309	0.256	0.565	0.0539	**Cause**	**2**
C3	0.192	0.300	0.493	−0.108	Effect	6
C4	0.318	0.265	0.583	0.052	**Cause**	**3**
C5	0.318	0.264	0.583	0.0541	**Cause**	**1**
C6	0.258	0.292	0.549	−0.034	Effect	5
**Mean**			**0.562**	**0.000**		
C1	0.118	0.187	0.304	−0.069	Effect	4
C2	0.288	0.126	0.414	0.162	**Cause**	**1**
C3	0.126	0.274	0.400	−0.149	Effect	6
C4	0.280	0.134	0.414	0.146	**Cause**	**2**
C5	0.174	0.156	0.331	0.018	**Cause**	**3**
C6	0.126	0.235	0.361	−0.108	Effect	5
**Mean**			**0.371**	**0.000**		

DM1 = Ukraine experts’ assessment; DM2 = Lithuania experts’ assessment.

Finally, the values of causality (Ri − Ci) and centrality (Ri + Ci) are calculated to represent influence-relation facts noticed after the multi-criteria analysis performed (see Table 5). Additionally, calculated causality (Ri − Ci) values are used to characterize the identity and rank of six resilience competencies for Ukrainian and Lithuanian soldiers.

The centrality results of this study disclosed the dissimilarities between the assessment of Ukrainian and Lithuanian experts. As a result of the evaluation of Ukrainian experts, the highest centrality value (D + R) appears for C1—self-awareness, C4—mental agility, and C5—strengths of character (see DM1, Table 5). The analysis of Lithuanian experts’ opinion conducted showed that the greatest value of centrality can be assigned to three resilience competencies, such as C2—self-regulation, C3—optimism, and C4—mental agility (see DM2, Table 5). The positive value in causality (D − R) was calculated for three resilience competencies: C2—self-regulation, C4—mental aversion, and C5—character strengths. But the values calculated to identify negative causality (D − R) let us identify the total similarity between the evaluation of Ukrainian and Lithuanian experts’ because the same resilience competencies were pointed out: C1—self-awareness, C3—optimism, and in this study, let us identify that C2, C4, and C5 are the criteria that play a causal role and influence C1, C3 and C6. Furthermore, the causal relationship analysis showed that according to Ukrainian experts, the C5 competence has the greatest influence on other resilience competencies, but for the opinion of Lithuanian experts, it is C2. Finally, the analysis conducted revealed that C6 is the most affected competence for both experts’ groups.

## 5 Discussion based on DEMATEL calculation results

The current study aimed to optimize a well-established military resilience program by identifying and retaining only the essential resilience competencies. While previous research have examined the importance of resilience competences in various military contexts including training (Sefidan et al., 2021; Bekesiene et al., 2022a), military missions (Carr et al., 2013), and combat operations (Haydabrus et al., 2022), they lacked an analysis of the importance of specific competencies in different stress contexts within the military. Our study filled this gap with a novel approach. Using the DEMATEL method, we conducted expert evaluations to determine the unique value of different competencies across diverse stress contexts.

Our findings reveal distinctive patterns: strengths of character (C5) emerged as most valuable in the context of combat operations, while self-regulation (C2) was vital in training and military missions. Significantly, our research results not only demonstrate that different stress contexts intensify the demand for distinct competencies (Van Wart and Kapucu, 2011), but also delineates these competencies in specific contexts. According to the findings, Ukrainian military experts, whose benchmark is conflicts within their own country, ranked the resilience competencies in the following order of importance: strengths of character (C5), self-regulation (C2), and mental agility (C4). Lithuanian experts, with more experience in performing military missions abroad, ranked resilience competencies in the following order of importance: self-regulation (C2), mental agility (C4), and strengths of character (C5).

**Figure 2:**
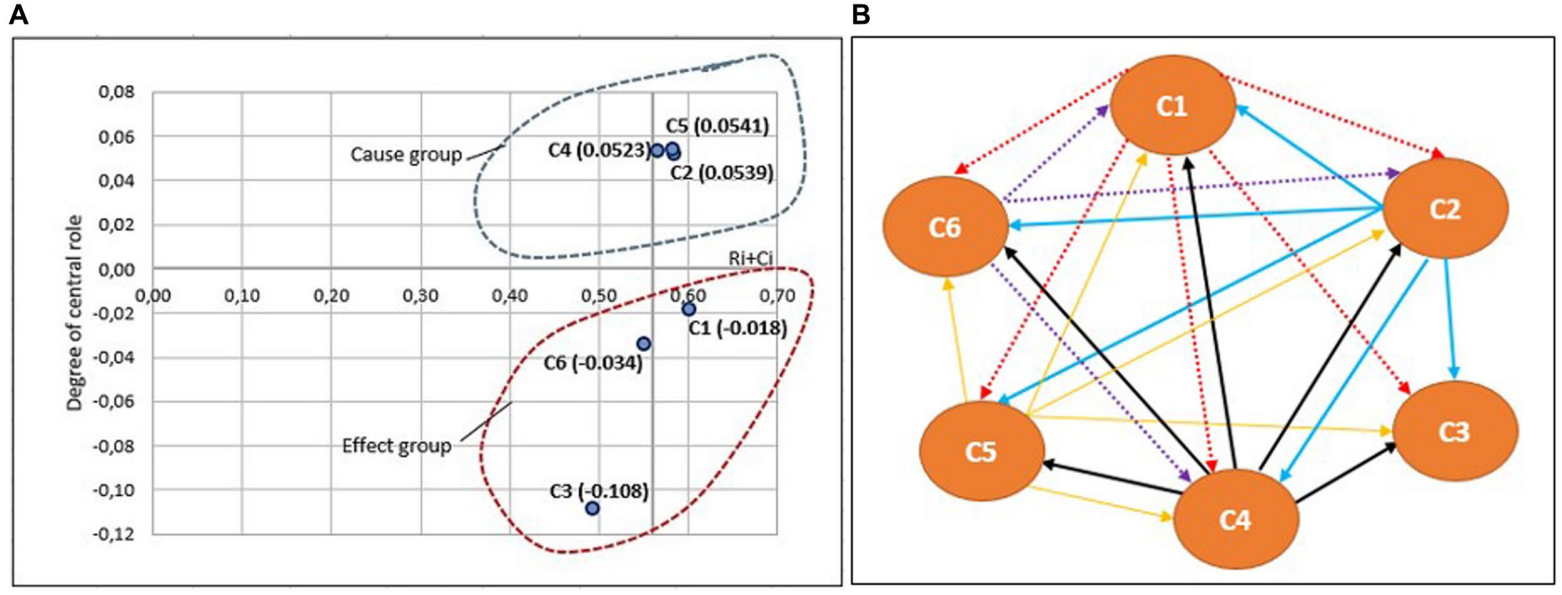
Graphical illustration of the structural model results based on Ukrainian soldiers’ opinion for six resilience competencies (C1 = self-awareness, C2 = self-regulation, C3 = optimism, C4 = mental agility, C5 = strengths of character, and C6 = connection): **(A)** a cause-and-effect diagram shows that C2, C4 and C5 are considered to be as causal factors, and C1, C3 and C6 are observed as an effect; **(B)** an influence-relation map between six resilience competencies after applied the threshold value =0.047.

**Figure 3:**
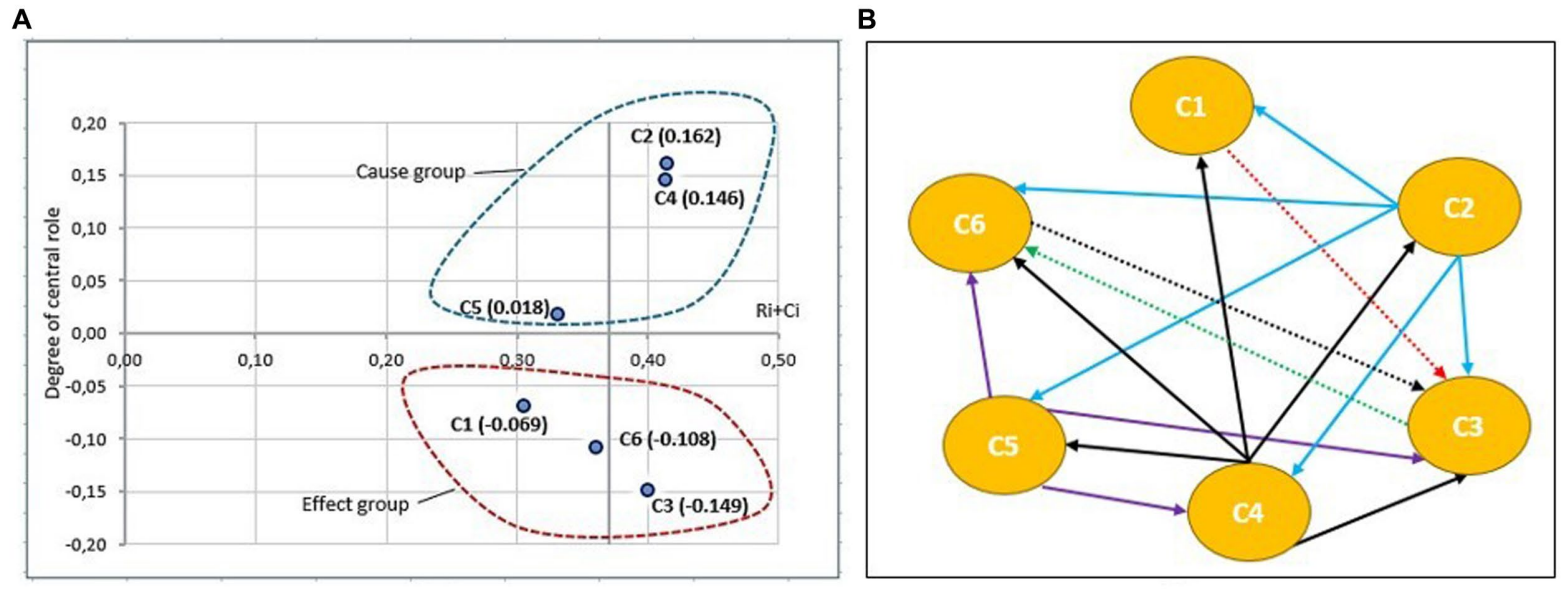
Graphical illustration of the structural model results based on Lithuanian soldiers’ opinion for six resilience competencies (C1 = self-awareness, C2 = self-regulation, C3 = optimism, C4 = mental agility, C5 = strengths of character, and C6 = connection): **(A)** a cause-and-effect diagram shows that R, M and S are considered to be as causal factors, and A, C and O are observed as an effect; **(B)** an influence-relation map between six resilience competencies an influence-relation map between six resilience competencies after applied the threshold value =0.031.

These findings require further elaboration. To achieve this, a DEMATEL causal relation diagram was employed to simplify intricate causal relationships into comprehensible graphic structures. Consequently, the diagram was divided into four quadrants based on the center points of the horizontal *X*-axis, which was set as prominence (R + C) and the vertical *Y*-axis, which was set as relation (R − C). These quadrants facilitated the simplification of identifying complex relationships among the six resilience competencies investigated while illustrating the influence of each competency on the others. Figure 2A presents the graphical representation of the designed structural model, depicting the results of the analysis of Ukrainian military experts on the six resilience competencies, while Figure 3A shows the results of the analysis of Lithuanian military experts.

Based on the four quadrants, the levels of mutual influence and causal relationships of the resilience competencies are categorized using prominence (R + C) and relation (R − C) values. This study enables us to identify differences in the assessment of psychological resilience competencies between two groups of experts. Ukrainian military experts, whose benchmark is conflicts within their own country, have a slightly different opinion compared to Lithuanian experts with a different experience (see Figures 2A, 3A). On the basis of the four quadrants, the following relationships were identified:

### 5.1 High relation and high prominence

Following the opinions of Ukrainian experts, three resilience competencies self-regulation (C2), mental agility (C4), and strengths of character (C5) were identified as crucial resilience competencies that influence and affect other resilience competencies. However, for Lithuanian experts, only two competencies, self-regulation (C2) and mental agility (C4), were considered vital for building resilience. These findings are in line with previous studies. Mental agility can be concluded to be a crucial competence of a soldier’s ability to perform effectively in stressful situations, involving quick and accurate information processing, critical thinking, and decision-making in high-pressure environments. Additionally, self-regulation is found to be not only a critical skill for soldiers to possess during stressful situations, but also increment the prediction of negative effects (McLarnon et al., 2021). Moreover, strengths of character were particularly important for Ukrainian experts, considering the challenging and often dangerous situations they encounter.

### 5.2 High relation and low prominence

According to the opinions of Lithuanian experts, the competence of character strengths (C5) in the cause group could influence several other resilience competencies, although not as strongly as indicated by the views of Ukrainian experts.

### 5.3 Low relation and high prominence

In this quadrant, the resilience competencies influenced by other competencies and not directly developable were identified. According to the opinions of Ukrainian experts, self-awareness (C1) fell into the category of effects, while the opinions of Lithuanian experts highlighted optimism (C3). Self-awareness is an important skill for soldiers to develop, as it helps them better understand their own strengths and weaknesses, cope with stress and adversity, and make better decisions in high-pressure situations.

### 5.4 Low relation and low prominence

Resilience competencies that fall into this structure were identified as relatively independent. Moreover, it can be noted that these competencies are influenced by other criteria, although to a lesser extent. The results of the analysis confirmed that for both groups of experts, the connection competence (C6) was relatively independent. However, there were differences regarding the other two resilience competencies: Ukrainian experts identified optimism (C3) as relatively independent, while Lithuanian experts considered self-awareness (C1) as such.

Although resilience is formed by a set of competencies, these competencies are found to be related to each other and not only complement each other, but also influence each other. This was highlighted by Luthar (2015) after an extensive overview of the existing literature on mental resilience. Based on the idea that competences affect each other, an influence-relation map based on the cause-and-effect relationship and mutual influence between the six main dimensions of resilience competencies illustrates the causal relationships between the dimensions of the resilience competencies of soldiers (see Figures 2B, 3B):

1. The influence-relation map based on the analysis of the Ukrainian experts’ dataset showed that optimism (C3) is the influencing factor affected by C1 (self-awareness), C2 (self-regulation), C4 (mental agility), and C5 (strengths of character). Additionally, C1 (self-awareness), C2 (self-regulation), C4 (mental agility), C5 (strengths of character), and C6 (connection) are interconnected, with inward-facing and outward-facing arrows indicating their influence and linkages. Furthermore, C2 (self-regulation), C4 (mental agility), and C5 (strengths of character) influence each other and are connect. Taking everything into account, the dominant factors influencing the improvement of resilience competencies to cope with stressful situations are C2 (self-regulation), C4 (mental agility), and C5 (strengths of character).
2. The influence-relation map based on the analysis of the Lithuanian experts’ dataset confirms that C1 (self-awareness), C2 (self-regulation), C3 (optimism), C4 (mental agility), C5 (strengths of character), and C6 (connection) are interconnected, with inward-and outward-facing arrows representing their influence and links. C3 (optimism) and C6 (connection) are related to each other. Moreover, C2 (self-regulation) and C4 (mental agility) influence and connect with each other, while C5 (strengths of character) influences C3 (optimism) and C6 (connection) and connects with C4 (mental agility). It appears that C2 (self-regulation) and C4 (mental agility) are the primary resilience competencies that should be included in Lithuanian soldiers’ resilience training programs.

Importantly, while the prioritization of competencies varies, the study reveals the consistent significance of three core competencies: strengths of character (C5), self-regulation (C2), and mental agility (C4). Notably, both groups of experts—those with a focus on conflicts within their own country and those experienced in military missions abroad—rated connection (C6) and optimism (C3) as the least important. This contradicts established academic literature (Iacoviello and Charney, 2020) and empirical studies of resilience (Schug et al., 2021), a highlighting the need for further investigation within military contexts.

## 6 Limitations and future research directions

Several limitations should be noted when interpreting the findings of this study. The first limitation could be related to the research results. Unlike other studies, the results of our study show that optimism and social connections are less significant than other resilience competencies. It may be attributed to the prevailing masculinity culture within the military, characterized by emotional detachment and self-control; resilience is better exemplified through self-regulation rather than social connections. Gueta and Shlichove (2022) research highlights that seeking assistance through social connections is viewed negatively, associated with weakness and femininity. These factors raise a discussion on the subjectivity of expert evaluations, as all evaluators were men, potentially influenced by their own stereotypes and identities (Wedgwood et al., 2022). Another aspect of masculinity, physical strength, as identified by Wedgwood et al. (2022), can be directly linked to our studied self-regulation, which experts may perceive as more “masculine” than optimism. Despite these potential stereotypes, the results of our study provide insight into how the scope of competencies developed for soldiers’ resilience can be narrowed in situations with limited time, focusing solely on the most critical competencies.

The second limitation of the study pertains to the country-specific military culture, as it solely focused on Ukrainian and Lithuanian soldiers. Considering that resilience training is deeply influenced by organizational culture, it is important to recognize the impact of country-specific organizational culture within the military as a variable that could have influenced research results. The significance of cultural differences has been widely acknowledged, particularly in the context of international military operations, see for example Yanakiev (2021).

Building on this understanding, the scope for future research becomes evident. First is to explore more deeply the cultural and gender influences on resilience assessments. The stereotypes and cultural norms prevalent among military professionals, as evidenced by the prevailing culture of masculinity identified in the study, raise intriguing questions. Investigating how these factors influence perceptions of resilience may provide a more comprehensive understanding.

Second, the identified competencies provide a basis for the design of targeted training programs. Future research should focus on the implementation and evaluation of these programs, analyzing their effectiveness in real military scenarios. In addition, exploring these competencies in different military branches and international contexts can further enrich the understanding of resilience requirements in different military environments.

## 7 Conclusion

This study contributed by adopting a reduction approach to identify key resilience competencies under the master resilience training scope, considering the time constraints faced by senior soldiers acting as trainers during deployment. Using the extended DEMATEL method, this study not only analyzed the evaluation perspectives and criteria of resilience training, but also established the cause-and-effect relationships among the competencies.

Research findings emphasize the importance of focusing on three essential resilience competencies: self-regulation, mental agility, and strengths of character. The specific significance of each of these competencies varies depending on the military context. In situations where conflicts persist within one’s own country, strengths of character emerge as the most influential competence for soldiers’ resilience. Conversely, in military operations conducted abroad, self-regulation plays a predominant role in fostering resilience.

This study stands out from previous scholarship by successfully applying the trapezoidal-fuzzy DEMATEL method to evaluate soldiers’ resilience competencies and categorize them into cause-and-effect groups. The results obtained offer valuable information for decision makers in improving the effectiveness of soldiers’ resilience training programs.

This study contributes to the theoretical understanding of military resilience competencies in several ways. First, the importance of resilience competencies is contextually differentiated. This study identifies distinct patterns in the demand for resilience competencies in two different stress contexts. Second, although resilience is a complex phenomenon, each resilience competency is attributed to a set of causes or effects. Such a cause-and-effect framework provides a better understanding of the links between competencies and enables researchers and practitioners to grasp the complexity of resilience in a structured way.

The findings of this study have practical implications for enhancing decision-making processes and improving the quality of soldiers’ resilience training programs. By focusing on the identified essential competencies, decision makers and trainers can tailor their approaches to effectively enhance the resilience of soldiers in the face of challenging military environments.

## Data availability statement

The raw data supporting the conclusions of this article will be made available by the authors, without undue reservation.

## Ethics statement

The study was approved by the General Jonas Zemaitis Military Academy, Protocol No. PR-1815. Informed consent was obtained from all subjects involved in the study.

## Author contributions

RK and SB: conceptualization and methodology. SB: software, formal analysis, resources, visualization, supervision, project administration, and funding acquisition. ON and SB: validation. RS, RV, ON, DB, and SB: writing for original draft preparation. SB, OK, and RS: writing for review and editing. All authors contributed to the article and approved the submitted version.
