# Supplementary material for: Prioritizing competencies for soldier’s mental resilience: an application of integrative fuzzy-trapezoidal decision-making trial and evaluation laboratory in updating training program
Source: Front Psychol. 2024 Feb 5;14:1239481. doi: 10.3389/fpsyg.2023.1239481 (PMC10875136; doi:10.3389/fpsyg.2023.1239481)
Supplement: Supplementary file 1 [file Data_Sheet_1.docx]

Supplementary Material

Prioritizing Competencies for Soldier's Mental Resilience: An Application of Integrative Fuzzy-Trapezoidal DEMATEL in Updating Training Program

Svajone Bekesiene*, Rasa Smaliukienė, Ramutė Vaičaitienė, Dalia Bagdžiūnienė, Rosita Kanapeckaitė, Olena Kapustyan and Oleksandr Nakonechnyi

*** Correspondence:** Svajone Bekesiene: svajone.bekesiene@lka.lt

# Supplementary Data Analysis Methodology: DEMATEL Extension by Trapezoidal Fuzzy Numbers

Extension of DEMATEL method by trapezoidal fuzzy numbers helps to perceive the uncertainty and diversity of sensations of linguistic decisions necessary to express essential associations (Hsu et al., 2007). In this study, experts were asked to express their opinions by labels related to a particular set of linguistic label set (Pribićević et al., 2020). So, nine linguistic variables were chosen to describe the assessment scores according to different criteria by linguistic variables: “Extremely low influence” (EXL), “Very low influence” (VLI), “Low influence” (LI), “Medium low influence” (MLI), “Medium influence” (MI), “Medium high influence” (MHI), “High influence” (HI), “Very high influence” (VHI), “Extremely high influence” (EXH) (see Table S1).

**Supplementary Table S1**. Trapezoidal fuzzy numbers and their relations to linguistic terms.

| **Linguistic terms (abbreviations)** | **Fuzzy trapezoidal values** | | | |
| --- | --- | --- | --- | --- |
|  | $\boldsymbol{a}_{\boldsymbol{1}}$ | $\boldsymbol{a}_{\boldsymbol{2}}$ | $\boldsymbol{a}_{\boldsymbol{3}}$ | $\boldsymbol{a}_{\boldsymbol{4}}$ |
| S1:Extremely low influence/(EXL) | (0, | 1, | 2, | 3) |
| S2:Very low influence/(VLI) | (1, | 2, | 3, | 4) |
| S3:Low influence/(LI) | (2, | 3, | 4, | 5) |
| S4:Medium low influence/(MLI) | (3, | 4, | 5, | 6) |
| S5:Medium influence/(MI) | (4, | 5, | 6, | 7) |
| S6:Medium high influence/(MHI) | (5, | 6, | 7, | 8) |
| S7:High influence/(HI) | (6, | 7, | 8, | 9) |
| S8:Very high influence/(VHI) | (7, | 8, | 9, | 10) |
| S9:Extremely high influence/(EXH) | (8, | 9, | 10, | 10) |

Note: Linguistic terms according to the fuzzy trapezoidal values (Chen-Yi et al., 2007).

This research provided analysis on the grouping procedure of trapezoidal fuzzy numbers and presented how linguistic terms $\left[ S_{l}, S_{u} \right]$ and $\left[ S_{\alpha}, S_{\beta} \right]$can be changed to a corresponding trapezoidal fuzzy number by using arithmetic operations and the membership function represented by Equation (1):

| $\mu_{\tilde{a}}(X)=\left\{ \begin{matrix} \frac{X-a_{1}}{a_{2}-a_{1}}, a_{1}\leq x\leq a_{2}, \\ \begin{matrix} 1, a_{2}\leq x\leq a_{3}, \\ \begin{matrix} \frac{a_{4}-X}{a_{4}-a_{3}}, a_{3}\leq x\leq a_{4}, \\ \begin{matrix} 0, & otherwise. \end{matrix} \end{matrix} \end{matrix} \end{matrix} \right.$ | (1) |
| --- | --- |

where $\tilde{a}$ is a fuzzy set of real numbers R and membership can be presented as $\tilde{a}:R\to[0,1]$, $x\in R\begin{matrix} , & \tilde{a} \end{matrix}\left( x \right)=1$. Therefore, the aggregation operations (addition ($\oplus)$, substraction (⊖), multiplication (⊗) and division (⊘)) between two linguistic terms ${[S}_{l,}S_{u}] and [S_{\alpha},S_{\beta}]$ can be expressed by the equations presented below:

${[S}_{l,}S_{u}]\oplus\left[ S_{\alpha},S_{\beta} \right]=\left( a_{lu}^{1}+a_{\alpha\beta}^{1},\cdots,a_{\alpha\beta}^{4}+a_{lu}^{4} \right)$ ; (2)

${[S}_{l,}S_{u}]⊖\left[ S_{\alpha},S_{\beta} \right]=\left( a_{lu}^{1}-a_{\alpha\beta}^{1},\cdots,a_{\alpha\beta}^{4}-a_{lu}^{4} \right)$; (3)

${k\otimes[S}_{l,}S_{u}]=\left( k\times a_{lu}^{1}, k\times a_{lu}^{2}, k\times a_{lu}^{3}, k\times a_{lu}^{4} \right)$; (4)

${{[S}_{l,}S_{u}]}^{-1}\cong\left( \frac{1}{a_{lu}^{4}}, \frac{1}{a_{lu}^{3}}, \frac{1}{a_{lu}^{2}}, \frac{1}{a_{lu}^{1}} \right)$. (5)

Consequently, the whole fuzzy-trapezoidal DEMATEL approach process can be completed after eight steps of analysis.

**Step 1.** At the beginning, we must design the direct – relation matrix $\hat{D}_{k}=\left[ \hat{d}_{ij} \right]_{nxn}$. First, the finite set of resilience competencies $C=\left\{ C_{1},C_{2},\cdots,C_{n} \right\}$ must be selected, where $C_{1}$ represents the *i*^th^ competence with $i\in\left\{ 1,2,\cdots,n \right\}$. Also, the set of skilled warriors can be used $W=\left\{ W_{1},W_{2},\cdots,W_{l} \right\}$, and $W_{k}$ characterises the $k^{th}$ warrior $k\in\left\{ 1,2,\cdots,l \right\}$. Consequently, to measure the study participants opinions a set of linguistic terms $T=\left\{ t_{0},t_{1},\cdots,t_{g} \right\}$, where $t_{g}$ represents the $s^{th}$ linguistic term, $s\in\left\{ 1,2,\cdots,g \right\}$ must be used, and as individually completed matrixes of warriors’ judgement must be collected. Accordingly, for each warrior $W_{k}$ , who was involved in the study, will design the direct relation matrices that can be presented by equation (6):

| $\hat{D}=\left[ \hat{d}_{kij} \right]_{n\times n}=\begin{matrix} C_{1} \\ \begin{matrix} C_{2} \\ \begin{matrix} \vdots\\ C_{n} \end{matrix} \end{matrix} \end{matrix}\left[ \begin{aligned} 0 \hat{d}_{k12} \cdots\hat{d}_{k1n} \\ \hat{d}_{k21} 0 \cdots\hat{d}_{k2n} \\ \vdots\vdots\ddots\vdots\\ \hat{d}_{kn1} \hat{d}_{kn2} \cdots0 \end{aligned} \right] ,$ | (6) |
| --- | --- |

where $k\in\{1,2,\cdots,l \}.$

**Step 2.** To continue the analysis procedure, the linguistic terms in the direct-relation matrices must be changed into trapezoidal fuzzy numbers following Equation (1). In case the participants of this study are said to be of equal significance, then the arithmetic mean of all of collected opinions can be used to design the main resilience competencies judgement matrix. To complete this procedure, arithmetic operations with trapezoidal fuzzy numbers must be conducted, and the matrix $\hat{D}=\left[ \hat{d}_{kij} \right]_{n\times n}$ must be transformed into $\tilde{D}=\left[ d \right]_{n\times n}$. In this way, following the arithmetic techniques offered by equations (2) and (4), all the direct-relation matrices $\hat{D}_{1}, \hat{D}_{2}, \cdots, \hat{D}_{k}$are aggregated into the key matrix.

**Step 3.** As a result, the set of uncertain direct- relation matrix $\tilde{D}=\left[ \tilde{d}_{kij} \right]_{n\times n}$ can be constructed. This procedure is focused on calculation of each $\tilde{d}_{kij}=\left( d_{kij}^{1},d_{kij}^{2},d_{kij}^{3},d_{kij}^{4} \right)$ element of the direct-relation matrix. This task can be completed by the following equations from (7a) to (7d):

| $d_{kij}^{1}=\frac{1}{n}\sum_{k=1}^{n} d_{kij}^{1}, i,j=1,2,\cdots,n;$ | (7a) |
| --- | --- |
| $d_{kij}^{2}=\frac{1}{n}\sum_{k=1}^{n} d_{kij}^{2}, i,j=1,2,\cdots,n;$ | (7b) |
| $d_{kij}^{3}=\frac{1}{n}\sum_{k=1}^{n} d_{kij}^{3}, i,j=1,2,\cdots,n;$ | (7c) |
| $d_{kij}^{4}=\frac{1}{n}\sum_{k=1}^{n} d_{kij}^{4}, i,j=1,2,\cdots,n.$ | (7d) |

**Step 4.** To continue the analysis procedure, the direct– relation matrix $\tilde{D}=\left[ \tilde{d}_{kij} \right]_{n\times n}$ must be transformed. So, next we will operate with the normalized indefinite direct-relation matrix $\tilde{Z}=\left[ \tilde{z}_{ij} \right]_{n\times n}$, and the each element $\tilde{z}_{ij}=\left( z_{ij}^{1},z_{ij}^{2},z_{ij}^{3},z_{ij}^{4} \right)$ of this matrix can be calculated by using the mathematical equations from (8a) to (8d):

| $z_{ij}^{1}=\frac{d_{ij}^{1}}{\max_{1\leq i\leq n}}\left\{ \sum_{j=1}^{n} d_{ij}^{1} \right\}, i,j=1,2, \ldots, n;$ | (8a) |
| --- | --- |
| $z_{ij}^{2}=\frac{d_{ij}^{2}}{\max_{1\leq i\leq n}}\left\{ \sum_{j=1}^{n} d_{ij}^{2} \right\}, i,j=1,2, \ldots, n;$ | (8b) |
| $z_{ij}^{3}=\frac{d_{ij}^{3}}{\max_{1\leq i\leq n}}\left\{ \sum_{j=1}^{n} d_{ij}^{3} \right\}, i,j=1,2, \ldots, n;$ | (8c) |
| $z_{ij}^{4}=\frac{d_{ij}^{4}}{\max_{1\leq i\leq n}}\left\{ \sum_{j=1}^{n} d_{ij}^{4} \right\}, i,j=1,2, \ldots, n.$ | (8d) |

Also, we must follow the main rule, which can be presented as follows.

| $\max_{1\leq i\leq n}\left\{ \sum_{j=1}^{n} m_{ij}^{4} \right\}\neq0, and 0 \leq z_{ij}^{1}\leq z_{ij}^{2}\leq z_{ij}^{3}\leq z_{ij}^{4}<1.$ | (9) |
| --- | --- |

In the following procedure, we will transform the normalized indefinite direct-relation matrix $\tilde{Z}$ into four crisp-value matrices $Z^{1}, Z^{2}, Z^{3}, Z^{4}$:

$Z^{1}=\left[ \begin{aligned} 0 z_{12}^{1} \cdots z_{1n}^{1} \\ z_{21}^{1} 0 \cdots z_{2n}^{1} \\ \vdots\vdots\ddots\vdots\\ z_{n1}^{1} z_{n2}^{1} \cdots0 \end{aligned} \right]$, $Z^{2}=\left[ \begin{aligned} 0 z_{12}^{2} \cdots z_{1n}^{2} \\ z_{21}^{2} 0 \cdots z_{2n}^{2} \\ \vdots\vdots\ddots\vdots\\ z_{n1}^{2} z_{n2}^{2} \cdots0 \end{aligned} \right]$,

$Z^{3}=\left[ \begin{aligned} 0 z_{12}^{3} \cdots z_{1n}^{3} \\ z_{21}^{3} 0 \cdots z_{2n}^{3} \\ \vdots\vdots\ddots\vdots\\ z_{n1}^{3} z_{n2}^{3} \cdots0 \end{aligned} \right]$, $Z^{4}=\left[ \begin{aligned} 0 z_{12}^{4} \cdots z_{1n}^{4} \\ z_{21}^{4} 0 \cdots z_{2n}^{4} \\ \vdots\vdots\ddots\vdots\\ z_{n1}^{4} z_{n2}^{4} \cdots0 \end{aligned} \right]$.

Where each of $\tilde{Z}^{k}$ can be calculated by using the multiplication procedure of crisp-value matrices.

**Step 5**. Now, the total-relation matrix $\tilde{G}$ , which can be presented by equation (10) must be designed:

| $\tilde{G}=\lim_{k\to+\infty} (\tilde{Z}^{1}\oplus\tilde{Z}^{2}\oplus\ldots\oplus\tilde{Z}^{k}); \tilde{G}=\left[ \tilde{g}_{ij} \right]_{n\times n}$ | (10) |
| --- | --- |

This procedure can be completed in a few steps. First, if we let matrix $\tilde{G}$ be represented as follows:

| $\tilde{G}=\left[ \begin{aligned} \tilde{g}_{11} \tilde{g}_{12} \cdots\tilde{g}_{1n} \\ \tilde{g}_{21} \tilde{g}_{22} \cdots\tilde{g}_{2n} \\ \vdots\vdots\ddots\vdots\\ \tilde{g}_{n1} \tilde{g}_{n2} \cdots\tilde{g}_{nn} \end{aligned} \right],$  $where \tilde{g}_{ij}=\left( g_{ij}^{1},g_{ij}^{2},g_{ij}^{3},g_{ij}^{4} \right),$ | (11) |
| --- | --- |

Then, the total-relation matrix can be constructed using the mathematical equations (12a) – (12d):

| $\left[ g_{ij}^{1} \right]_{n\times n}=G^{1}\left( I-G^{1} \right)^{-1}, i,j=1,2,\cdots,n;$ | (12a) |
| --- | --- |
| $\left[ g_{ij}^{2} \right]_{n\times n}=G^{2}\left( I-G^{2} \right)^{-1}, i,j=1,2,\cdots,n;$ | (12b) |
| $\left[ g_{ij}^{3} \right]_{n\times n}=G^{3}\left( I-G^{3} \right)^{-1}, i,j=1,2,\cdots,n;$ | (12c) |
| $\left[ g_{ij}^{4} \right]_{n\times n}=G^{4}\left( I-G^{4} \right)^{-1}, i,j=1,2,\cdots,n.$ | (12d) |

**Step 6**. Starting from this step we focus on the identification of the total strengths of influencing and the influenced relationship of resilience competencies $C_{1},C_{2},\cdots,C_{n}$ that were contained within analysis. Therefore, we must calculate the sum of each row ($\tilde{r}_{i}=(r_{i}^{1},r_{i}^{2},r_{i}^{3},r_{i}^{4})$ of matrix $\tilde{G}$, and the sum of each column $\tilde{c}_{i}=(c_{i}^{1},c_{i}^{2},c_{i}^{3},c_{i}^{4})$ of matrix $\tilde{G}$ . This procedure helps identify the global intensity in which the resilience competence $C_{n}$ is influenced by others.

**Step7**. Consequently, in case to determine the uncertain variance and correlation of each resilience competencies the sum of $\tilde{r}_{i}$ and $\tilde{c}_{i}$ must be calculated by using the mathematical equations from (13a) to (13d):

| $s_{i}^{1}=r_{i}^{1}+c_{i}^{1}, i=1,2, \ldots, n;$ | (13a) |
| --- | --- |
| $s_{i}^{2}=r_{i}^{2}+c_{i}^{2}, i=1,2, \ldots, n;$ | (13b) |
| $s_{i}^{3}=r_{i}^{3}+c_{i}^{3}, i=1,2, \ldots, n;$ | (13c) |
| $s_{i}^{4}=r_{i}^{4}+c_{i}^{4}, i=1,2, \ldots, n.$ | (13d) |

Moreover, the relationships among the resilience competencies investigated can be identified according to the difference between each of $\tilde{r}_{i}$ and $\tilde{c}_{i}$. These differences can be calculated by using the mathematical equations from (14a) to (14d):

| $d_{i}^{1}=r_{i}^{1}-c_{i}^{1}, i=1,2, \ldots, n;$ | (14a) |
| --- | --- |
| $d_{i}^{2}=r_{i}^{2}-c_{i}^{2}, i=1,2, \ldots, n;$ | (14b) |
| $d_{i}^{3}=r_{i}^{3}-c_{i}^{3}, i=1,2, \ldots, n;$ | (14c) |
| $d_{i}^{4}=r_{i}^{4}-c_{i}^{4}, i=1,2, \ldots, n.$ | (14d) |

**Step 8.** The last step is focused on the determination of the crisp importance and relation of each resilience competence. This procedure can be completed by using the centroid (centre of gravity) measures (Yager et al., 1994) that can be calculated by equations (15) and (16):

| $s_{i}=\frac{1}{4}\left( s_{i}^{1}+s_{i}^{2}+s_{i}^{3}+s_{i}^{4} \right)$; | (15) |
| --- | --- |
| $d_{i}=\frac{1}{4}\left( d_{i}^{1}+d_{i}^{2}+d_{i}^{3}+d_{i}^{4} \right)$. | (16) |

In addition, on calculated $s_{i}$ and $d_{i}$ values can be drawn a causal diagram to visualize the importance and specifically classification of the investigated resilience competencies. To explain complex causal relationships, all resilience competencies are divided into understandable visual structures that help distinguish the category of each competency according to its place in the diagram.
